# Autoimmune disorders but not heparin are associated with cell-free fetal DNA test failure

**DOI:** 10.1186/s12967-018-1705-2

**Published:** 2018-12-03

**Authors:** Yohann Dabi, Sarah Guterman, Jacques C. Jani, Alexandra Letourneau, Adèle Demain, Pascale Kleinfinger, Laurence Lohmann, Jean-Marc Costa, Alexandra Benachi

**Affiliations:** 1 Service de Gynécologie-Obstétrique, AP-HP, Hôpital Antoine Béclère, Université Paris Sud, 154 rue de la Porte de Trivaux, 92140 Clamart, France; 2 Human Genetics Department, Laboratoire CERBA, Saint-Ouen l’Aumône, France; 3 Department of Obstetrics and Gynecology, University Hospital Brugmann, Université Libre de Bruxelles, Brussels, Belgium

**Keywords:** Autoimmune disorder, Cell-free DNA screening, Heparin treatment, Non-reportable result, Prenatal, Noninvasive, Systemic lupus erythematosus

## Abstract

**Background:**

Recent studies have suggested a possible association between heparin treatment at the time of cell-free DNA (cfDNA) testing and a non-reportable result. However, these studies lack of proper methodology and had a low level of proof to firmly incriminate heparin. Our objective was to investigate further the relationship between heparin treatment and cfDNA test results.

**Methods:**

Two complementary approaches were used for the demonstration. First, we conducted a retrospective analysis of a cohort of patients with a singleton pregnancy, screened for aneuploidies by using cfDNA, but with a non-reportable cfDNA result. We included patients between 2013 and 2016 including the patients from the DEPOSA study as controls. CfDNA testing was performed by massive parallel sequencing by using a whole-genome approach. A multiple logistic regression was used to account for the influence of the variables included. Second, we performed in vitro experiments on mimic samples containing increased concentrations of heparin.

**Results:**

Of 9867 singleton pregnancies tested during the inclusion period, 58 (0.59%) had a non-reportable result and were compared to 295 control patients. Fifteen (25.9%) and 20 (6.8%) patients were treated with heparin in the group with a non-reportable cfDNA result and with a successful assay, respectively. In multivariable analysis, an increased calculated risk at the first-trimester combined screening (OR 28.8 CI 9.76–85.15, p < 0.001), maternal weight (OR 1.03, CI 1.01–1.06, p = 0.01), and the presence of an autoimmune disease (OR 10.38, CI 1.62–66.53, p = 0.01) were the only characteristics associated with a non-reportable result. In vitro experiments showed that heparin had no impact on fetal fraction measurement or the final result, no matter what the dose tested.

**Conclusions:**

Treatment by heparin had no impact on cfDNA screening test for aneuploidies, while the presence of an autoimmune disorder is an independent predictor of a non-reportable result.

## Background

Since Lo et al. first demonstrated the presence of fetal DNA sequences in maternal plasma and serum [1], many improvements have been made to clinical application of this tool. Cell-free DNA (cfDNA) screening for aneuploidy is now widely used in most Western countries and its wide-ranging utility keeps increasing over the years. Many studies show that cfDNA screening has a higher detection rate (> 99%) and a lower false-negative rate (< 1%) than any other screening approach available so far [2–4].

Discrepancies are reported regarding the rate of cfDNA non-reportable results in cfDNA, probably because in many cases it is not clear whether the authors are reporting failure for a single sample or after a second sample analysis [5, 6]. The latest review by Gil et al. shows that clinicians experience test failure in up to 4% of cases [7].

The most common reason for non-reportable results is a fetal fraction below 4%, which is the usual cut-off for decision making [8–10]. Such results delay both decision-making and invasive procedures. Parameters affecting fetal fraction include maternal and fetal characteristics such as ethnicity, body mass index, smoking, modes of conception, as well as fetal chromosomal anomalies [11–13]. Recent reports of low fetal fractions in patients treated with low-molecular-weight heparin (LMWH) raised the question of the impact of treatment on test results [14–16]. However, with few patients included and no proper design, those studies could neither confirm nor reject this hypothesis.

Based on reports that in vitro heparin has no effect on cfDNA analysis [17, 18], we hypothesized that heparin could not be held responsible for cfDNA test failure and that there were confounding factors that might be involved in the aforementioned cases of reported failures. We used two complementary approaches to investigate further the relationship between heparin treatment and cfDNA results.

## Methods

### Cohort analysis

From November 2013 to March 2016, 9867 patients carrying a singleton pregnancy underwent cfDNA screening in regular clinical practice. The indications for testing were those currently accepted in the French national recommendations [19] i.e. mainly maternal age 38 or older at delivery, prior pregnancy with trisomy, positive test result (risk > 1/250) for aneuploidy with maternal serum screening, including first-trimester combined screening or parental balanced Robertsonian translocation with increased risk of fetal trisomy 13 or trisomy 21. In some cases, cfDNA screening was performed for patients with ultrasound findings, as accepted by the American College of Obstetrics and Gynecology [20] and by the Society for Maternal–Fetal Medicine [21] and as a first-line screening test. Some of those patients were patients from the DEPOSA study as this study was prospective and interventional and results were given to the patients and used for clinical management [3].

Patients with a non-reportable cfDNA result at first blood sample were included in the study. Their characteristics were retrieved from their charts and phone call interviews were used to collect pregnancy outcomes. Their characteristics were compared with those of patients from the control group. This group was constituted with patients included in the DEPOSA study [3]. For this latter group, complete information regarding medical condition prior to and during pregnancy and with pregnancy follow-up was available.

Patients with multiple pregnancies were excluded, as were those with incomplete data or missing information regarding treatment received and/or medical condition prior to pregnancy. Heparin doses in patients treated could be either prophylactic or curative.

In line with French regulations regarding prenatal diagnosis, written informed consent was obtained from all patients as the result was used for clinical management. Laboratoire CERBA is authorized by the Regional Health Agency to perform these screening tests. Regarding patients who participated in the DEPOSA study, our local institutional review board approved this study (CPP No. 14-054) (ClinicalTrials.gov number, NCT02424474).

### Cell-free fetal DNA analysis in maternal plasma

Analysis was performed by massive parallel sequencing (MPS) by using a whole-genome approach as described elsewhere with slight modifications [22]. Maternal blood was collected in two cfDNA BCT Streck^®^ tubes (10 mL each) and sent at + 4 °C to the clinical lab where plasma was isolated within 4 days after collection by a double centrifugation procedure and stored frozen at ≤ − 70 °C if not processed immediately. Total DNA was extracted from 4 mL of plasma with the QIAamp DSP Circulating Nucleic Acid Kit (Qiagen, Courtaboeuf, France). DNA library preparations were sequenced in an Illumina V3 flow cell on a HiSeq1500 instrument with the Truseq SBS kit V3-HS reagent (Illumina, Paris, France) for 27 cycles followed by 7 or 8 cycles to read each sample index. Finally, sequence reads were mapped to the UCSC hg19 version of the human genome using Bowtie version 2, Z-scores were calculated for the targeted chromosomes 13, 18 and 21 as described and classification was based upon a standard normal transformed cutoff value of z = 3 for chromosome 21 and z = 3.95 for chromosomes 18 and 13. As part of the assay, the fetal fraction was estimated using the sequence read approach (SeqFF) described by Kim et al. [23]. This “non-fetal-specific” method is based on a multivariate regression model and on subtle fragment length differences and the inferred non-uniformity of fetal cfDNA across the genome.

Results are expressed as “positive” or “negative” when the metric criteria are fulfilled (library concentration 7.5 nM or greater, total count of reads ≥ 9 million and the estimated fetal DNA fraction ≥ 4%). Results were expressed as non-reportable when the estimated fetal DNA fraction was less than 4% or when Z-scores were positive for more than one chromosome.

### In vitro testing for heparin impact on cfDNA assay

To mimic samples collected in patients treated with heparin, a pool of 100 plasma samples (NPP) previously tested as negative for cfDNA screening was processed with escalating doses of either enoxaparin or tinzaparin. These LMWH molecules were chosen as the most frequently used in France. LMWH doses were calculated to reproduce clinical situations i.e. a low dose for patients with prophylactic treatment, a medium dose as curative dose and a high dose for overdose situations, so 0.6, 1, 2 and 2.4 IU/mL for enoxaparin and 0.43, 0.87 and 1.74 IU/mL for tinzaparin. These mimic samples were then treated in the same manner as for sample patients in our routine process (88 samples per flow cell experiment). Results were compared to the same NPP tested in quadruplicate during the same experimental run. Experiments were performed twice.

### Statistical analysis

All statistical analysis was performed by a statistician (JJ) that was completely independent of the data collection.

Statistical analysis was based on Student’s t test for continuous variables and the χ^2^ test or Fisher’s exact test for categorical variables. Univariable logistic regression analysis was used to investigate the effect on test failure rate of patients treated with LMWH (yes, no), patients with autoimmune disease (yes, no), method of conception (IVF, non-IVF), high risk for Down syndrome of maternal serum screening (yes, no), as categorical variables and maternal age (years), gestational age at test (in weeks), maternal weight (in kg) as continuous numerical variables. Auto-immune diseases included systemic lupus erythematosus (SLE), antiphospholipid syndrome (APL) and Hashimoto’s thyroiditis. The parameters we chose to include in the model were those usually reported to be associated with a non-reportable test result in the literature. Multiple logistic regression analysis was subsequently performed to determine the significant independent contribution of those variables yielding a *p *< 0.05 in the univariate analysis.

Data were analyzed using the statistical software SPSS, version 24.0 (Chicago, Illinois, USA), and Excel, version 9.0 (Microsoft, Redmond, Washington, USA). A two-sided p-value of less than 0.05 was considered statistically significant.

## Results

### Cohort analysis

**Fig. 1:**
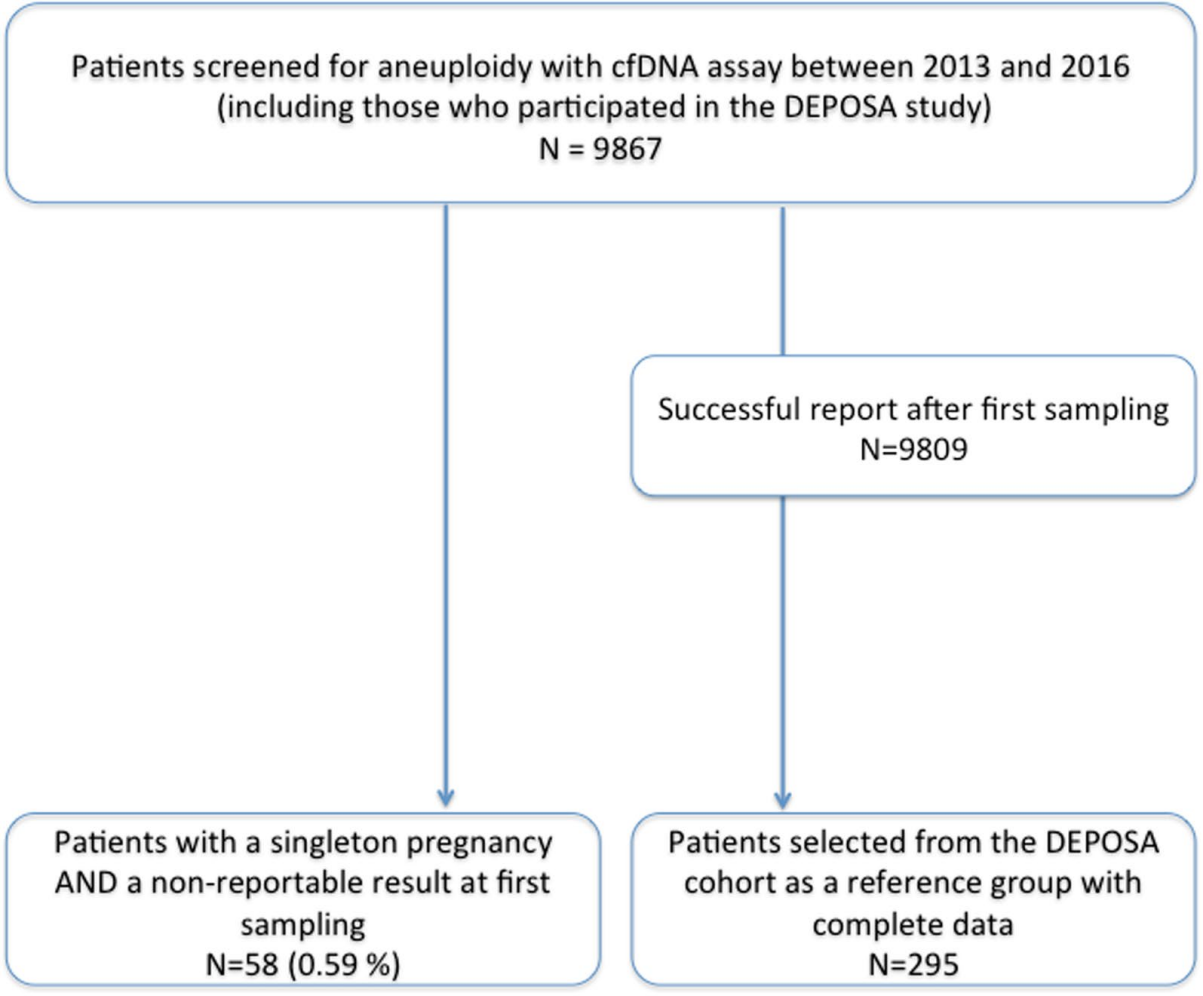
Flow chart of the study

Between January 2013 and December 2016, 58 (0.59%) patients had cfDNA test failure at first blood sample and were included (Fig. 1) along with 295 control patients.

Patients with no test results were significantly older, had higher BMI and were more often nulliparous (p < 0.001) (Table 1). Seven patients (12.1%) in the group of patients with a non-reportable cfDNA result had an autoimmune disorder prior to the pregnancy, compared with only 5 (1.7%) patients with a successful assay. Fifteen patients (25.9%) were treated with heparin at the time of cfDNA screening with a non-reportable cfDNA result and 20 patients (6.8%) with a successful assay. The causes of a non-reportable result at the first sample were as follows: low fetal fraction (32 patients, 55.2%) and atypical z-scores (26 patients, 44.8%). Forty-eight patients had a second test where the results showed a second non-reportable result in 11 patients (22.9%). The main outcomes of the pregnancies are presented in Table 2. Patients had pre-eclampsia and intrauterine growth retardation (< 0.001) significantly more often than their counterparts with successful cfDNA testing.

**Table 1:** Main characteristics of the patients included

	cfDNA not reportable n = 58	cfDNA successful n = 295	p-value
Maternal age (years)	37 (35.7–40.1)	34 (29.6–37.1)	< 0.001
Maternal weight (kg)	77 (60–90.3)	64 (56–70)	< 0.001
Maternal height (cm)	165 (161.8–171)	165 (161–169)	0.53
BMI	29 (22.3–32.9)	23.7 (20.7–25.9)	< 0.001
Ethnic group			0.14*
Caucasian	41 (70.7)	237 (80.3)	
Asian	1 (1.7)	13 (4.4)	
Black or Caribbean	7 (12.1)	41 (13.9)	
Other/unknown	9 (15.5)	4 (1.4)	
Cigarette smoker	4 (6.9)	1 (0.3)	
Nulliparous	22 (37.9)	18 (6.1)	< 0.001
History of aneuploidy	3 (5.2)	9 (3.1)	
Medical condition			
Autoimmune disease	7 (12.1)	5 (1.7)	< 0.001
Repeated vein thrombosis	8 (13.8)	3 (1.0)	< 0.001
Other chronic condition	5 (8.6)	35 (11.9)	0.65
Heparin received	15 (25.9)	20 (6.8)	< 0.001
Antiplatelet therapy	9 (15.5)	24 (8.1)	0.11
Any other treatment received^a^	14 (24.1)	75 (25.4)	0.96
Mode of conception			0.83
Spontaneous	50 (86.2)	248 (84.1)	
Assisted	8 (13.8)	47 (15.9)	
Reason for referral			
First-trimester screening	40 (70.0)	35 (11.9)	
Second-semester screening	10 (17.2)	1 (0.3)	
Maternal age	4 (6.9)	0	
History of aneuploidy	4 (6.9)	1 (0.3)	
Ultrasound abnormalities	0	19 (6.4)	
First line screening	0	239 (81.0)	

Data are given as mean (interquartile range) or n (%)

1 patient had a history of medical abortion for EDFR2 mutation

* Caucasian versus other ethnic group

^a^Including: nicardipine/hydroxychloroquine/digoxin/corticosteroids/chloroquine/levothyroxine

**Table 2:** Pregnancy outcomes

	Cases n = 58 (%)	Controls n = 295 (%)	p-value
Complications of pregnancy			
Pre-eclampsia	8 (13.8)	3 (1.0)	< 0.001
IUGR < 5th percentile	6 (10.3)	8 (2.7)	< 0.001
Fetal death	2 (3.4)	0	
Other^a^	11 (19.0)	78 (26.4)	0.3
Infant			
Weeks of gestation at birth	37.2 (36.6–40)	38 (38.4–40.4)	0.36
Weight (in grams)	2856 (2423–3566)	3139 (2900–3552)	0.02
Size	48.9 (47.3–52)	48.9 (48–50)	0.96
Fetal malformation	2 (3.4)	4 (1.4)	0.26

^a^Includes any other complication that occurred during the pregnancy (diabetes, premature rupture of membranes…)

Univariable logistic regression analysis demonstrated that significant predictors of non-reportable results were maternal age (p < 0.001), gestational age at cfDNA screening (p < 0.001), maternal weight (p < 0.001), increased risk for Down syndrome after serum screening (p < 0.001), treatment with LMWH (p < 0.001) and auto-immune disorders (p < 0.001). In multivariable analysis, an increased calculated risk at first-trimester combined test screening (OR: 28.8, CI 9.76–85.15, p < 0.001), maternal weight (OR: 1.03, CI 1.01–1.06, p = 0.01) and the presence of an autoimmune disease (OR: 10.38, CI 1.62–66.53, p = 0.01) were the sole characteristics associated with a non-reportable result. The remaining parameters were not independently associated with an increased risk of non-reportable results at first sampling for cfDNA screening (Table 3).

**Table 3:** Logistic regression to predict non-reportable results of cfDNA testing

Variable	N (%) or median (range)	Non-reportable cfDNA assay			
		Univariate analysis		Multivariate analysis	
		OR (95% CI)	p	OR (95% CI)	p
Maternal age (years)	34 (18–49)	1.105 (1.046–1.167)	< 0.001	1.006 (0.926–1.094)	0.883
Gestational age (weeks)	12.6 (11.1–27.5)	1.674 (1.436–1.953)	< 0.001	1.136 (0.936–1.380)	0.198
Maternal weight (kg)	63 (43–110)	1.046 (1.028–1.065)	< 0.001	1.034 (1.006–1.063)	*0.017*
High risk for down syndrome in maternal serum screening^a^					
Yes	60 (20.1)	48.364 (21.319–109.714)	< 0.001	28.823 (9.757–85.146)	< *0.001*
No	239 (79.9)	1		1	
IVF					
Yes	51 (16.1)	0.639 (0.273–1.499)	0.304		
No	266 (83.9)	1			
Patients treated with LMWH					
Yes	35 (11.0)	4.555 (2.176–9.535)	< 0.001	1.993 (0.539–7.377)	0.301
No	282 (89.0)	1		1	
Patients with autoimmune disease					
Yes	12 (3.8)	9.731 (2.825–33.528)	< 0.001	10.376 (1.618–66.526)	*0.014*
No	305 (96.2)	1		1	

Italic values indicate significance of p value (p <  0.05)

*LMWH* low-molecular-weight heparin, *IVF* in vitro fertilization

^a^Missing data in 18 cases

### In vitro testing for heparin impact on cfDNA assay

For every sample tested, there was no significant difference regarding the library prep concentration, the number of reads or fetal fraction measurement with or without addition of LMWH, no matter the molecule used at any concentration. Overall results are summarized in Table 4. All samples tested had a fetal fraction > 4% even after addition of a high dose of LMWH and values observed were similar to those for the equivalent samples not supplemented with LMWH and the mean value observed for the entire flow cell experiment. Furthermore, there was no impact on the calculated Z-score for the three autosomal chromosomes leading to the same final reportable interpretation. Such results were obtained in two distinct experiments.

**Table 4:** Summarized results of in vitro testing for heparin impact on cfDNA assay

Experiment	Sample	Heparin	Molecule	Concentration IU/mL	Library prep (nM)	Reads (M)	Fetal fraction (%)	Z-score 21	Z-score 18	Z-score 13
1	NPP	No	–	–	26.16	20.1	11.7	− 0.78	− 1.55	0.97
1	NPP	No	–	–	31.01	17.0	10.91	0.92	− 0.85	0.01
1	NPP	No	–	–	32.29	16.0	10.32	− 0.72	0.00	− 0.42
1	NPP	No	–	–	30.79	18.9	10.03	− 0.44	− 0.08	− 0.48
1	Entire flow cell	No	–	–	31.10	18.0	10.20	0.00	0.04	0.01
1	NPP	Yes	Enoxaparin	0.6	27.05	18.8	10.80	0.36	0.80	− 0.01
1	NPP	Yes	Enoxaparin	1.2	34.37	18.5	11.10	1.51	0.20	0.24
1	NPP	Yes	Enoxaparin	2.2.44	23.89	18.7	11.90	0.31	− 0.58	0.45
1	NPP	No	–	–	22.40	19.9	11.25	− 0.75	− 0.49	− 0.76
1	NPP	No	–	–	26.28	19.7	10.53	− 0.34	0.22	− 0.20
1	NPP	No	–	–	29.70	17.0	11.08	− 0.72	− 1.07	− 0.84
1	NPP	No	–	–	32.84	19.2	11.34	− 0.35	0.04	− 0.46
1	Entire flow cell	No	–	–	30.98	18.8	9.80	0.02	0.00	0.02
1	NPP	Yes	Tinzaparin	0.43	27.05	21.6	9.40	− 1.21	0.42	1.49
1	NPP	Yes	Tinzaparin	0.87	34.37	21.2	9.50	− 0.17	0.04	− 1.43
1	NPP	Yes	Tinzaparin	1.74	23.89	19.7	10.00	− 0.34	− 0.02	0.24
2	NPP	No	–	–	38.95	18.6	8.97	0.67	− 0.02	− 2.15
2	NPP	No	–	–	37.99	18.5	10.32	− 1.17	1.16	− 0.16
2	NPP	No	–	–	35.89	17.4	11.86	− 1.34	− 0.06	− 0.05
2	NPP	No	–	–	36.63	18.2	11.85	− 0.10	− 0.72	− 0.73
2	Entire flow cell	No	–	–	33.40	18.2	10.35	0.06	0.08	0.06
2	NPP	Yes	Enoxaparin	0.6	37.98	18.9	11.30	0.68	− 0.26	1.25
2	NPP	Yes	Enoxaparin	1.2	32.51	19.9	10.40	0.15	0.15	− 0.61
2	NPP	Yes	Enoxaparin	2.2.44	36.92	20.3	11.50	0.35	− 0.37	− 1.03
2	NPP	No	–	–	22.33	19.8	10.26	0.43	0.66	0.56
2	NPP	No	–	–	24.38	17.7	10.25	− 0.15	− 0.76	0.51
2	NPP	No	–	–	26.56	17.5	9.93	− 0.10	0.21	1.21
2	NPP	No	–	–	28.83	18.0	10.44	0.70	− 0.47	− 0.99
2	Entire flow cell	No	–	–	29.00	18.2	10.30	− 0.01	0.03	− 0.07
2	NPP	Yes	Tinzaparin	0.43	32.24	22.2	10.50	0.37	0.92	0.68
2	NPP	Yes	Tinzaparin	0.87	33.72	19.2	9.50	1.03	0.59	0.56
2	NPP	Yes	Tinzaparin	1.1.7474	32.06	20.5	10.30	0.19	1.12	0.54

*NPP* normal plasma pool, entire flow cell: mean data obtained within the same experiments for the 88 samples. Z-score 21: Z-score observed for chromosome 21, Z-score 18: Z-score observed for chromosome 18, Z-score 13: Z-score observed for chromosome 13

## Discussion

We report here a large cohort of patients undergoing cfDNA testing with a non-reportable result at the first attempt. We chose to evaluate non-reportable results after first sampling because these failures represent one of the most critical clinical management issues. The rate of no-result at first sampling is low (0.59%) as it was in our previous studies [3, 24].

This study is the first to demonstrate the effect of a pre-existing medical condition i.e., an autoimmune disorder, on the rate of non-reportable cfDNA assays. In our cohort, 12 patients had a pre-existing autoimmune disease and most of them required heparin treatment, which is a common feature shared with various studies [14–16, 25]. Our controlled study shows that heparin cannot be solely held responsible for test failure.

As heparin is often part of treatment of autoimmune diseases, some authors wrongly hold it responsible for such test failure. In the present study, we investigated whether heparin treatment in pregnant women could impact the final result of cfDNA screening for aneuploidy. Studies that have suggested a relationship between heparin and the rate of non-reportable cfDNA results have not considered the underlying conditions for which heparin therapy was indicated, i.e. autoimmune diseases [14–16]. Grömminger et al. analyzed a set of 5 patients with non-reportable results after a first sample and performed a second cfDNA test just before the next injection of heparin [15]. This was the first study to examine a potential impact of heparin on cfDNA results. They concluded that pregnant women on LMWH had a higher proportion of small DNA fragments featuring an unusually high guanosine–cytosine (GC) content, which could potentially influence the measurements of cfDNA. On the other hand, Burns et al. suggested that LMWH results in low fetal fractions, due to reduced fetal DNA release into the maternal circulation because heparin reduces trophoblast apoptosis [16]. In another recent study, Ma et al. [14] concluded that the use of LMWH reduced the fetal fraction and rendered cfDNA false-negative in their single case report, while the fetus was carrying trisomy 21, even if the rate of aneuploidy is increased among patients with non-reportable results [26]. Our in vitro study cannot rule out this latter hypothesis, but it does show that LMWH itself does not interfere with the analysis. Neither the type nor the dose of heparin used had an impact on fetal fraction measurement and the samples were all successfully analyzed. This is in line with a recent report showing that retrieved cell-free DNA is protected from the pre-analytical impact of blood DNase in EDTA plasma [17]. Similar results have been reported with heparin plasma [18]. While the results of our in vitro experiment are clear, one cannot exclude that a third agent might interfere in vivo on the effect of heparin on placenta, which could be considered as a limitation of our study.

As we do not fully understand the origin of cell-free fetal DNA [27], the factors involved in no-call results in cfDNA testing are incompletely elucidated. Autoimmune disorders like SLE or APL could affect cfDNA results either by distorting cfDNA measurement or by impacting the fetal fraction. Many authors have reported increased levels of serum total DNA in patients with active SLE [28, 29], which could lead to a low fetal fraction. On the other hand, in pregnant patients with SLE, increased syncytiotrophoblast apoptosis secondary to SLE/APL could increase the overall quantity of cfDNA. Such patients usually have impaired kidney function and Hui et al. reported increased fetal fraction in patients following immunosuppressive treatment [25]. In our cohort, 32 patients with non-reportable results had a fetal fraction below 4% and the remaining ones had atypical results with a positive z-score for more than one chromosome. To the best of our knowledge, only one study has compared cfDNA in patients with and without SLE and found that, in the third trimester, these two groups had a similar quantity of fetal DNA [30]. Besides the lack of determinant data regarding fetal fraction, third-trimester data are less relevant to the understanding of the impact of SLE/APL on cfDNA results. Further studies should focus on comparing these two populations in early pregnancy. Patients with SLE show a remarkable expression of anti-DNA antibodies and we hypothesize that this could lead to plasma DNA modifications.

cfDNA screening has been developed and validated in a mostly healthy population, and our study questions its applicability to specific groups. We emphasize here the need for clinicians to exercise care when analyzing cfDNA test results, especially non-reportable results in specific populations. We recognize that our analysis has some limitations. We restricted our inclusions to patients with singleton pregnancies with complete information available for the main clinical and biochemical factors usually associated with increased risk of non-reportable results. Therefore, we only included patients when information on heparin treatment and medical condition at the time of cfDNA testing was available (patients from the DEPOSA study). Furthermore, the amount of first and second line screening is not equal in the non reportable group and in the control group, with more first line tests in the control group, but, at least in our hands, the cfDNA test works equally in both situations [3, 24] We are aware that this might have introduced bias, but our multivariable analysis of the factors involved in test failure is consistent with the literature and so is a strong indicator of the validity of our results. While we demonstrated that heparin could not be held solely responsible for non-reportable results in those patients, we were not able to assess the impact of other treatments on cfDNA testing. It is theoretically possible that other treatment commonly used in patients with auto-immune disorders (such as steroids for example) negatively impacted cfDNA testing as well. Hui et al. reported increased fetal fraction in a patient with a history of severe autoimmune thrombocytopenia following the introduction of an immunosuppressive treatment by steroids [31]. Such reports raise the question of the impact of treatment on cfDNA testing and further studies should focus on understanding this complex relationship.

## Conclusion

Our study ruled out the hypothesis that heparin treatment has an impact on cfDNA screening and found that autoimmune diseases are associated with test failure. A limitation of our work lies within its retrospective nature and further studies with larger samples and prospective design should help improve our knowledge of the factors involved in non-reportable test result.
